# Differential responses of crop pollen microbial communities to insect visitation and host identity: fungi are more sensitive than bacteria

**DOI:** 10.3389/fmicb.2026.1789970

**Published:** 2026-03-23

**Authors:** Yage Li, Bo Wang, Congcong Lan, Panfeng Dai, Lina Zhao, Mingming Zhang, Qiang Fang

**Affiliations:** 1 College of Agriculture, Henan University of Science and Technology, Luoyang, China; 2 College of Food and Bioengineering, Henan University of Science and Technology, Luoyang, China

**Keywords:** community assembly, cross-pollination, host plant identity, insect visitation, pollen microbiome

## Abstract

**Introduction:**

In the pollination process, pollen serves not only as a key vehicle for plant reproductive success but also as an important ecological interface for microbial transmission and selection. However, how insect visitation and host plant identity jointly affect the assembly of pollen microbial communities in agroecosystems remains poorly understood.

**Methods:**

Here, we employed bagging and open-pollination treatments combined with high-throughput sequencing to investigate the effects of insect visitation and host plant identity on the structure, composition, diversity, interspecific interactions, and core taxa of pollen microbial communities in crops.

**Results:**

Results showed that insect visitation and host plant identity jointly and significantly influenced the structure of pollen bacterial asnd fungal communities and altered their taxonomic composition, diversity, and interaction patterns, with these effects being mainly evident in cross-pollinated plants and more pronounced in fungal than in bacterial communities. Further analyses revealed that insect visitation increased network connectivity while reducing modularity (0.046) in bacterial communities of cross-pollinated plants, whereas their fungal network exhibited reduced connectivity and increased modularity (0.787). In cross-pollinated plants, fungal core taxa dominated the fungal communities (>87%), while bacterial core taxa contributed relatively little to overall community.

**Dicussion/conclusion:**

Overall, insect visitation and host plant identity jointly shape pollen microbial communities, but bacterial and fungal communities exhibit distinct response patterns, with bacterial communities being relatively stable and fungal communities being more sensitive. This study highlights the key roles of insect visitation and host plant identity in pollen microbiome assembly and provides a theoretical basis for understanding crop pollination ecology and plant–pollinator–microbe interactions.

## 1 Introduction

Mutualistic interactions between plants and pollinators play a crucial role in maintaining biodiversity, ecosystem stability, and agricultural production (Potts et al., 2010; Katumo et al., 2022). Within this interaction system, flowers not only serve as sites for the exchange of resources such as pollen and nectar but also function as important interfaces for the transmission of signals, including floral scent and color. Recent studies have shown that diverse and abundant microbial communities inhabit floral surfaces and secretions (Junker and Keller, 2015; Rering et al., 2018). These microbes are deeply involved in plant–pollinator interactions by influencing pollen germination and viability (Christensen et al., 2021), altering the physicochemical properties of nectar (Rering et al., 2018), and modulating pollinator visitation behavior (Russell and Ashman, 2019). Meanwhile, as flower-visiting insects move among flowers, they not only facilitate pollen transfer but also promote the dispersal of microorganisms among flowers and plant individuals, acting as important biological vectors linking plants and pollinators (Russell et al., 2019; Olson et al., 2023). Consequently, flower-associated microorganisms are increasingly recognized as key “ecological regulators” that can influence host health, pollinator behavior, and ultimately the outcomes of plant–pollinator interactions (Vannette, 2020; Steffan et al., 2024).

Currently, the composition and diversity of floral microbial communities vary with differences in flower-visiting insect identity, host plant identity, and floral sex (Zemenick et al., 2021; Marre et al., 2022). In addition to whole flowers, microorganisms have also been detected in floral components such as anthers, nectar, stigmas, styles, and petals (Junker and Keller, 2015; Olson et al., 2023). Among these, nectar has received particular attention (Cusumano et al., 2023; Deng et al., 2024), and its microbial composition and functions have been shown to be regulated by host plant identity (Fridman et al., 2012), pollination type (Jacquemyn et al., 2013), and flower-visiting insect identity (de Vega et al., 2021), thereby exerting positive, neutral, or negative effects (Rering et al., 2018; Deng et al., 2024) on plant–pollinator interactions. In contrast, although pollen is also an important floral reward for pollinators and rich in nutrients, existing studies have largely focused on its role as a food resource for pollinators or human health (Kieliszek et al., 2018; Dharampal et al., 2019), whereas our understanding of pollen microbial communities and their ecological significance remains relatively limited.

Studies have shown that the composition and diversity of pollen microbial communities are influenced by factors such as year, geographic distribution, host plant identity, and pollination type, and that bacterial and fungal communities exhibit markedly different responses to environmental variation (Ambika Manirajan et al., 2018; Armstrong et al., 2024). Even within the same geographic region, the microbial composition of pollen can differ significantly among plant species or cultivars (Ambika Manirajan et al., 2018; Khalaf et al., 2023). In addition, both beneficial microorganisms and potential pathogens have been detected in pollen (Kim et al., 2018; Shrestha et al., 2024). However, these studies are largely based on samples collected under natural conditions and lack controlled experiments, such as bagging treatments, that explicitly isolate the effects of insect visitation. Consequently, the potential contribution of insects to the assembly and dynamics of pollen microbial communities remains difficult to quantify.

In fact, flower-visiting insects have been demonstrated to be important vectors of flower-associated microorganisms (Russell et al., 2019; Olson et al., 2023), and these microbes may play differentiated roles at different stages of the pollination process, thereby influencing pollination success (Cullen et al., 2021; Steffan et al., 2024). Nevertheless, the specific effects of insect visitation on the composition and dynamics of pollen microbial communities remain poorly understood, particularly in agroecosystems that are highly dependent on insect pollination, where relevant studies are still limited. Therefore, experimental designs that control for insect visitation are essential to clarify the roles of insect visitation and host plant identity in pollen microbiome assembly, which is of critical importance for understanding plant–pollinator–microbe interaction mechanisms in agricultural ecosystems.

Agained this background, we investigated a range of insect-pollinated and wind-pollinated crops and applied bagging and open-pollination treatments to systematically examine the joint effects of insect visitation and host plant identity on pollen microbial communities. We hypothesized that: (1) insect visitation would significantly reshape the structure of pollen microbial communities and influence their taxonomic composition and diversity; (2) pollen microbial communities of plants with different pollination types would exhibit distinct response patterns to insect visitation; and (3) insect-mediated pollen-associated microorganisms, particularly core taxa, play critical roles in maintaining community structure and potential ecological functions within plant–pollinator–microbe interactions. This study aims to elucidate the assembly characteristics of pollen microbial communities in the context of pollination activities and to provide an ecological perspective for a deeper understanding of plant–pollinator–microbe interaction mechanisms in agricultural ecosystems.

## 2 Materials and methods

### 2.1 Study sites and experimental design

This study was conducted from July to August 2024 in Yanglou Town, Ruzhou City, Henan Province, China (34.1815°N, 112.6890°E). The study area is characterized by flat terrain, fertile soils, and intensive agricultural activity. Four common vegetable crops were selected as study species: *Capsicum annuum* L. (CA), *Solanum melongena* L. (SM), *Luffa aegyptiaca* Mill. (LA), and *Momordica charantia* L. (MC). All crops were planted at the same time and managed under identical agricultural practices, including irrigation, fertilization, and pesticide application. The four crop species were planted in adjacent plots within the same experimental field, ensuring consistent microclimatic conditions across species and treatments. CA and SM are monoecious species with bisexual flowers and are predominantly self-pollinated. Insect visitation can enhance their pollination, and the observed flower visitors were mainly bees, but at relatively low frequencies. In contrast, LA and MC are monoecious species with unisexual flowers and rely on flower-visiting insects for cross-pollination; observed visitors included bees, flies, and ants. Therefore, based on their predominant pollination systems, CA and SM were classified as self-pollinated hosts, whereas LA and MC were classified as cross-pollinated hosts in this study.

For each plant species, two treatments were applied: open (natural pollination) and bagging (exclusion of most flower-visiting insects) treatments. A number of intact, unopened flower buds were randomly selected and assigned to the two treatments. In the bagging treatment, buds were enclosed in mesh bags (mesh size approximately 0.4 mm), whereas buds in the open treatment were marked with tags only. All pollen samples were collected from freshly opened flowers within the same anthesis window to ensure consistency in floral age and pollen maturation stage across treatments. Flowers of all four plant species opened during the daytime and typically reached full anthesis at approximately 06:00 in the morning. After 10 h of exposure following flower opening, flowers enclosed in intact mesh bags and tightly secured to the pedicel and the marked flowers were collected. Their pollen was harvested using sterile forceps and centrifuge tubes. Due to substantial differences in pollen production among plant species, the number of flowers pooled per sample varied among species, ranging from 10 to 80 flowers, to obtain sufficient pollen for DNA extraction and sequencing. Within each species, the number of flowers pooled per sample was kept consistent between open and bagging treatments to avoid treatment-specific bias. Given that the objective of this study was to assess community-level differences rather than individual-flower variation, pooling multiple flowers per sample provided a more representative estimate of pollen microbial communities at the plant species level. For each species, five replicates were established for each treatment, resulting in a total of 40 pollen samples, which were stored at −80 °C until further processing.

In addition, pollen from a single flower was collected from both the open and bagging treatments for each of the four plant species. Petals were carefully removed using forceps, and all anthers were excised and placed into centrifuge tubes containing 1 mL of absolute ethanol. Pollen grains were then thoroughly washed off the anthers by centrifugation. For each plant species and each treatment, five replicates were established, yielding a total of 40 single-flower pollen samples, which were stored at 4 °C. Pollen grains were subsequently counted using a standard light microscope and a hemocytometer.

### 2.2 High-throughput sequencing

Pollen samples stored at −80 °C were thawed under cold conditions, after which subsamples were homogenized, mechanically ground, and centrifuged. Total genomic DNA was extracted using the QIAGEN DNeasy PowerSoil Pro Kit following the manufacturer’s instructions. DNA concentration and purity were assessed with a NanoDrop 2000 spectrophotometer, and DNA integrity was verified by electrophoresis on 1% agarose gels. The extracted DNA was used as a template for amplification of the bacterial 16S rRNA gene and the fungal internal transcribed spacer (ITS) region. Bacterial amplification was performed using primers 799F (AACMGGATTAGATACCCKG) and 1193R (ACGTCATCCCCACCTTCC) (Chelius and Triplett, 2001; Bodenhausen et al., 2013), while fungal amplification employed primers ITS1F (CTTGGTCATTTAGAGGAAGTAA) and ITS2R (GCTGCGTTCTTCATCGATGC) (White et al., 1990; Gardes and Bruns, 1993). PCR reactions were conducted in 20 μL volumes using Pro Taq polymerase. Amplification products were checked on 2% agarose gels, purified, and used for library construction, followed by paired-end sequencing (PE300) on the Illumina MiSeq platform. During PCR amplification, no-template PCR controls were included to monitor potential contamination, and no visible amplification bands were detected in control samples. Negative extraction controls and mock community standards were not incorporated in this study. Raw paired-end reads generated from sequencing were first quality-filtered to remove adapter sequences and low-quality reads using the UPARSE pipeline (v7.0.1090), resulting in clean reads. Clean reads were then merged and screened for chimeric sequences using UCHIME implemented in UPARSE. After chimera removal, the remaining high-quality sequences were defined as valid reads. Valid reads were clustered into operational taxonomic units (OTUs) at 97% sequence similarity. Representative OTU sequences were selected for taxonomic assignment using the RDP Classifier against the SILVA database for bacteria^1^ and the UNITE database for fungi.^2^ OTUs classified as chloroplast or mitochondria during taxonomic annotation were identified as host-derived sequences and removed prior to all downstream analyses. All diversity analyses, community composition analyses, and network analyses were performed based on the resulting host-filtered valid reads. A total of 2,536,412 bacterial valid reads and 2,717,167 fungal valid reads were retained for downstream analyses, corresponding to 3,920 bacterial OTUs and 1,076 fungal OTUs, respectively. High-throughput sequencing of pollen microbiomes was carried out by Shanghai Majorbio Bio-pharm Technology Co., Ltd. (Shanghai, China).

### 2.3 Co-occurrence network analyses and core taxa

Based on pollination type and treatment groups, CA and SM were categorized into self-pollinated open (Self_open) and self-pollinated bagging (Self_bagging) groups, whereas LA and MC were categorized into cross-pollinated open (Cross_open) and cross-pollinated bagging (Cross_bagging) groups, to compare differences in pollen microbial co-occurrence patterns among treatment combinations. Co-occurrence networks of pollen bacteria, fungi, and bacteria–fungi interactions were constructed using genus-level relative abundance data based on Spearman’s rank correlation matrices. Given that bacterial and fungal communities differ substantially in abundance distributions, occurrence frequencies, and data sparsity (Faust and Raes, 2012), different filtering strategies were applied to ensure network robustness while minimizing the exclusion of ecologically relevant taxa. Bacterial genera were retained if they occurred in at least five samples and had a mean relative abundance ≥ 0.05% across samples. In contrast, fungal communities exhibited lower overall abundance and higher sparsity; therefore, a less restrictive occurrence threshold was applied to avoid excessive removal of fungal taxa. Preliminary sensitivity analyses indicated that more stringent thresholds substantially reduced fungal network connectivity and complexity. Accordingly, fungal genera were retained if they had a mean relative abundance ≥ 0.001% across samples. For both bacterial and fungal networks, only correlations with an absolute Spearman’s correlation coefficient (|*ρ*|) ≥ 0.65 were retained, and statistical significance (*p*-value) was adjusted using the Benjamini–Hochberg method to control the false discovery rate (FDR) (*q* < 0.05). For bacteria–fungi interaction networks, only cross-domain correlations meeting the above criteria were retained. Topological properties were then calculated for all co-occurrence networks.

Because network size and density differed among groups, we followed the theoretical framework proposed by Banerjee et al. (2018) to identify core taxa (keystone genera) based on node degree and betweenness centrality. Specifically, nodes ranked within the top 10% for either degree or betweenness centrality were defined as core taxa. Alternative selection rules were evaluated during preliminary analyses; requiring nodes to rank within the top 10% for both degree and betweenness centrality resulted in no core taxa being identified in some smaller networks. Therefore, the proportional (top 10%) criterion based on degree or betweenness centrality was retained to ensure comparability among networks of different sizes. Functional prediction of bacterial and fungal communities was performed using PICRUSt2^3^ and FUNGuild,^4^ respectively, and the relative abundance of predicted functions was calculated at the genus level. Within each group, the taxonomic relative abundances and predicted functional relative abundances of all core genera were first summed for each sample. The mean values and standard errors (SE) of these two metrics were then calculated across samples within each group, allowing the overall relative contributions of core taxa to the community to be quantified from both taxonomic and predicted functional perspectives.

### 2.4 Statistical analyses

For the bacterial 16S rRNA dataset, following quality control, chimera removal, and removal of host-derived OTUs (chloroplast and mitochondria), sequencing depth across the 40 samples ranged from 12,317 to 90,581 valid reads (mean = 64,072 reads; Supplementary Table S1). One sample exhibited markedly lower sequencing depth (12,317 reads) compared with the remaining samples and was therefore excluded from alpha (*α*) diversity analyses to avoid substantial information loss that would have resulted from rarefaction to an excessively low threshold. The remaining bacterial samples were rarefied to 40,000 valid reads per sample prior to *α* diversity analysis. This threshold was selected to retain all remaining samples while minimizing data loss. For the fungal ITS dataset, sequencing depth after quality control and chimera removal ranged from 39,307 to 170,134 valid reads (mean = 67,929 reads). No OTUs were taxonomically assigned to chloroplast or mitochondria in the fungal dataset; therefore, no host-derived OTUs were removed. All fungal samples exceeded the rarefaction threshold and were therefore retained for downstream analyses. Fungal samples were rarefied to 39,000 valid reads per sample prior to *α* diversity analysis. Although sequencing depth varied substantially among fungal samples, rarefaction ensured comparability across samples. For beta diversity analyses, Bray–Curtis dissimilarities were calculated based on relative abundance data derived from the non-rarefied OTU tables after removal of host-derived OTUs. Bray–Curtis dissimilarities based on relative abundances are relatively robust to variation in sequencing depth, and thus rarefaction was not applied. Co-occurrence network analyses were also constructed using relative abundance data from the same non-rarefied OTU tables to preserve information on taxon occurrence frequency and abundance patterns across samples.

At the phylum level, the relative abundances of bacterial and fungal communities in pollen were calculated for the four crop species under the two treatments. For each plant species, Venn analyses were conducted to identify shared and unique OTUs between the open and bagging treatments, and the numbers and proportions of these OTUs were summarized. At the OTU level, *α* diversity indices, including Chao, Shannon, and Faith’s phylogenetic diversity (PD), were calculated. Two-way ANOVA and *F*-tests were used to evaluate the main effects of treatment and plant species, as well as their interaction, on *α* diversity indices. When significant main effects or interactions were detected, Welch’s ANOVA followed by Games–Howell *post-hoc* tests (*p* < 0.05) were applied to compare *α* diversity among different treatments and plant species. Based on Bray–Curtis dissimilarities, non-metric multidimensional scaling (NMDS) was employed to assess beta diversity patterns of bacterial and fungal communities among treatments and plant species, and differences among groups were tested using permutational multivariate analysis of variance (PERMANOVA).

The mean pollen quantity of the bagging treatment for each crop species was used as a baseline, and pollen remaining rate (%) in the open treatment was calculated as (pollen quantity in the open treatment / mean pollen quantity in the bagging treatment) × 100, which served as a proxy for insect visitation frequency. Differences in pollen remaining rates among species were first tested for homogeneity of variances and then analyzed using one-way ANOVA followed by Tukey’s HSD *post-hoc* tests (*p* < 0.05). Because pollen samples used for microbial sequencing and those used for pollen quantity measurements were not paired, species-level analyses were conducted using the mean relative abundance of total predicted functions of core taxa in the open treatment across the four crop species to examine the relationship between pollen remaining rate and the total predicted functions of core taxa. Given the relatively small sample size in the open treatment, a bootstrap-based species-level Spearman correlation analysis was applied for bacteria: within each species, five replicates were resampled with replacement (bootstrap), species means were calculated for each resampling, and Spearman’s *ρ* was then computed to obtain the distribution of *ρ* and the corresponding 95% confidence intervals (CI). For fungi, because core taxa were detected only in the cross-pollinated open treatment, Cohen’s d effect size was used to compare differences between LA and MC.

All statistical analyses and data visualizations in this study were performed using R version 4.5.2, SPSS 22.0, Gephi 0.10.1, and OriginPro 2024.

## 3 Results

### 3.1 Microbial community structure

**Figure 1:**
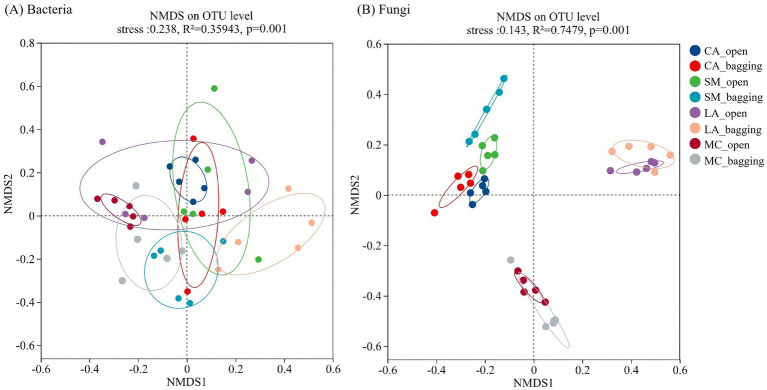
NMDS ordination of pollen bacterial and fungal community composition among plant species based on Bray–Curtis dissimilarities. **(A)** bacterial communities and **(B)** fungal communities. CA, *Capsicum annuum* L.; SM, *Solanum melongena* L.; LA, *Luffa aegyptiaca* Mill.; MC, *Momordica charantia* L.

NMDS analyses showed significant differences in the composition of pollen bacterial and fungal communities among plant species under the open and bagging treatments (Figure 1). Specifically, 35.94% of the variation in bacterial communities could be explained by grouping factors such as treatment and plant species, representing a moderate level of explained variation, and this effect was significant (*R*^2^ = 0.35943, *p* = 0.001); however, partial overlap among groups was observed (Figure 1A). In contrast, grouping factors including species and treatment explained as much as 74.79% of the variation in fungal communities, with a highly significant effect (*R*^2^ = 0.7479, p = 0.001). The fungal communities of CA and SM did not overlap but were positioned relatively close to each other, and were completely separated from those of LA and MC (Figure 1B). Venn diagram analysis further revealed pronounced differences in OTU shared and unique patterns between bacterial and fungal communities under the two treatments (Supplementary Figure S1). Bacterial communities exhibited higher numbers and proportions of shared OTUs, whereas fungal communities showed lower shared OTU numbers and proportions. Under the open treatment, the proportion of unique fungal OTUs reached 75.23 and 77.14% in LA and MC, respectively, while the corresponding proportions under the bagging treatment were only 6.82 and 7.27% (Supplementary Figure S1). Overall, bacterial communities maintained higher proportions of shared OTUs across treatments, whereas fungal communities in cross-pollinated host plants exhibited higher proportions of unique OTUs under the open treatment.

**Figure 2:**
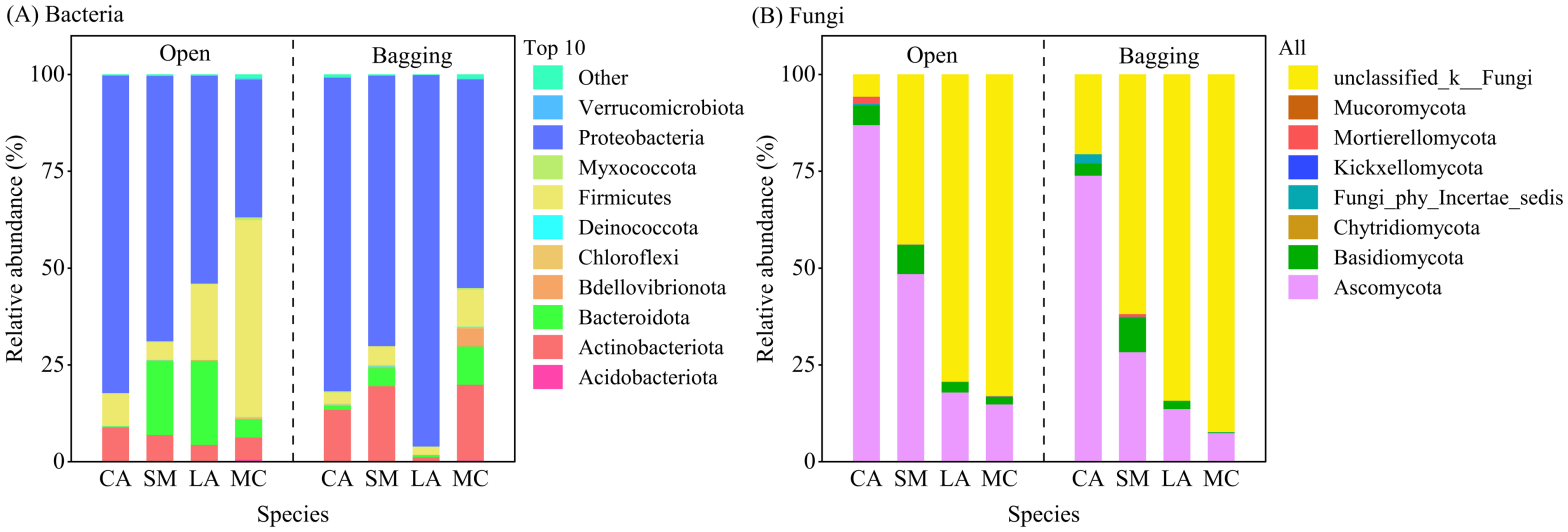
Relative abundance of bacterial and fungal phyla in pollen. **(A)** bacterial communities and **(B)** fungal communities.

Across the 40 pollen samples, a total of 35 bacterial phyla, comprising 1,099 genera, and 8 fungal phyla, comprising 374 genera, were detected (Figure 2). In bacterial communities, the dominant phylum was Proteobacteria (35.53–95.92%, mean 67.51%), followed by Firmicutes (2.10–50.89%), Bacteroidota (0.27–21.78%), and Actinobacteriota (1.16–19.53%), whereas the remaining 31 phyla each exhibited mean relative abundances below 1% (Figure 2A). In fungal communities, excluding unclassified fungi, Ascomycota was the dominant phylum (7.42–87.02%, mean 36.46%), followed by Basidiomycota (0.19–8.84%); the remaining five phyla each accounted for less than 1% of the mean relative abundance (Figure 2B). The open treatment introduced additional fungal phyla, including Chytridiomycota in SM and LA and Kickxellomycota in LA and MC. Compared with the bagging treatment, the open treatment resulted in the introduction or loss of several low-abundance phyla depending on plant species. For example, the low-abundance bacterial phylum TX1A-33 and fungal phyla Kickxellomycota and Mucoromycota were detected in the open treatments of LA and MC, whereas low-abundance bacterial phyla (MBNT15, Sva0485, Thermotogota, and Zixibacteria), as well as fungal phylum Mucoromycota were not detected in the open treatment of CA. Overall, both treatment and plant species jointly influenced the taxonomic composition and relative abundance patterns of pollen microbial communities.

### 3.2 Microbial community alpha diversity

**Figure 3:**
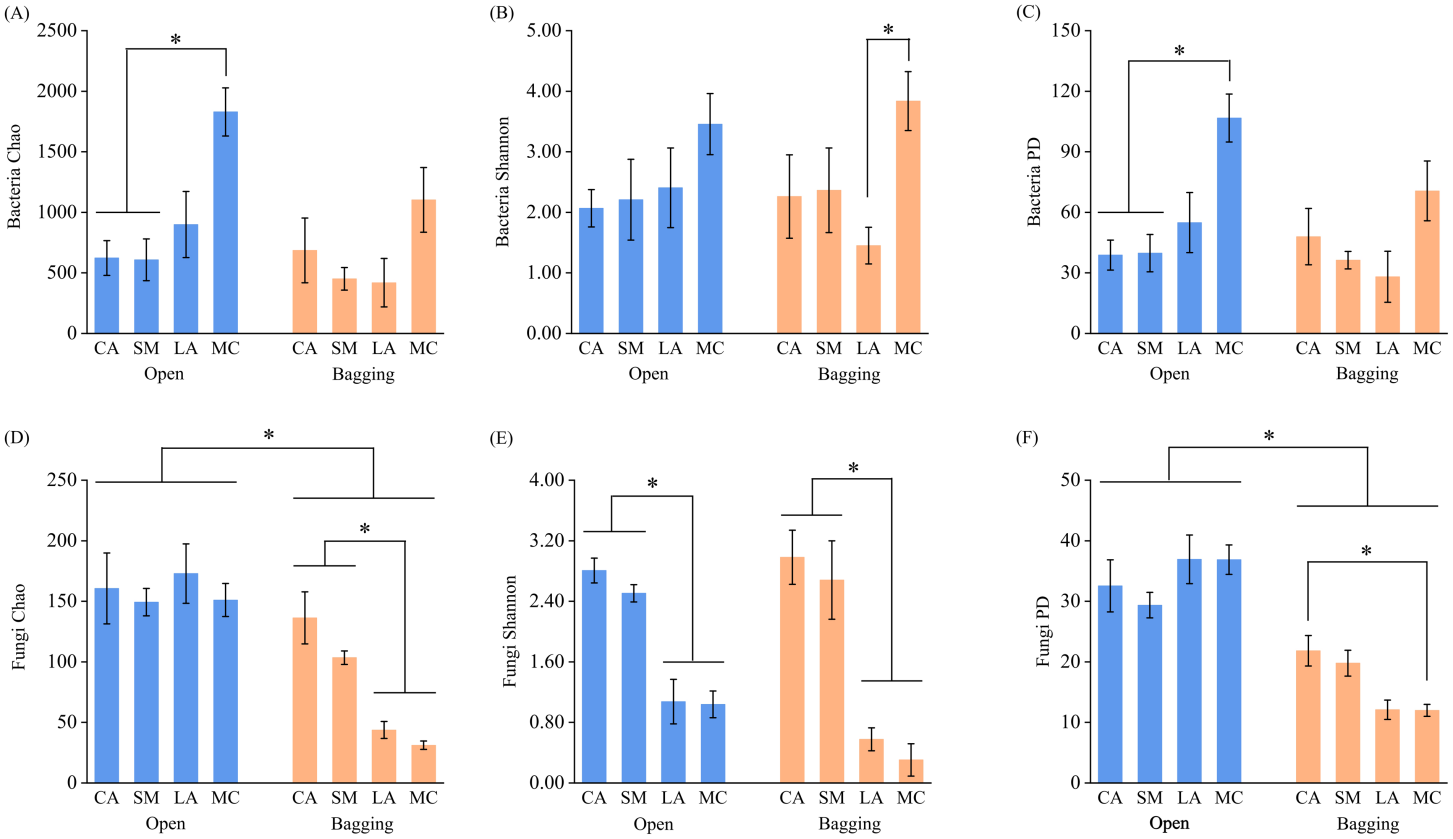
Differences of pollen bacterial and fungal *α* diversity among different treatments and plant species. *, *p* < 0.05. **(A)** Bacteria Chao index; **(B)** Bacteria Shannon index; **(C)** Bacteria PD index; **(D)** Fungi Chao index; **(E)** Fungi Shannon index; **(F)** Fungi PD index.

The ANOVA results showed that treatment significantly affected the Chao index of both bacterial and fungal communities, as well as the PD index of fungal communities, whereas plant species significantly influenced the Chao and Shannon indices of both bacterial and fungal communities and the PD index of bacterial communities (Supplementary Table S2). Under the open treatment, the bacterial Chao and PD indices of MC were significantly higher than those of CA and SM, while the fungal Shannon indices of LA and MC were significantly lower than those of CA and SM (*p* < 0.05). Under the bagging treatment, the fungal Chao and Shannon indices of LA and MC were significantly lower than those of CA and SM, and the fungal PD index of MC was significantly lower than that of CA (*p* < 0.05) (Figure 3). Compared with the bagging treatment, the open treatment led to increases in the fungal Chao, Shannon, and PD indices in LA and MC (Figures 3D–F). Overall, the interaction between treatment and plant species had significant effects only on the fungal Chao and PD indices, whereas no significant interaction effects were observed for bacterial diversity indices (Supplementary Table S2; Figure 3).

### 3.3 Bacterial-fungal co-occurrence network

Co-occurrence network analysis was used to explore interactions within pollen bacterial and fungal communities. Across the eight single bacterial and fungal networks, positive interactions accounted for more than 97% of all significant correlations. Notably, no significant negative correlations were detected in the bacterial network of the self-pollinated open treatment, the fungal network of the self-pollinated bagging treatment, and the fungal network of the cross-pollinated bagging treatment (Supplementary Figure S2, Supplementary Table S3). The bacterial network under the cross-pollinated open treatment exhibited a more generalized structure, with very low modularity (modularity = 0.046), whereas the remaining seven networks all displayed clear modular structures (modularity > 0.41). Among bacterial single networks, the cross-pollinated open network showed the highest complexity and connectivity, characterized by the largest numbers of nodes, edges, and core taxa, the highest average degree, clustering coefficient, and network density, as well as the shortest average path length (Supplementary Table S3). In fungal single networks, compared with the bagging treatment, networks under the open treatment also exhibited larger network size, with increased numbers of nodes and edges. In particular, relative to the cross-pollinated bagging treatment, the fungal network under the cross-pollinated open treatment showed a pronounced increase in modularity, with the numbers of modules and core taxa increasing by approximately threefold (Supplementary Table S3). Overall, fungal single networks showed consistently higher clustering coefficients, smaller network diameters, and shorter average path lengths than bacterial single networks, while containing a greater number of modules.

**Figure 4:**
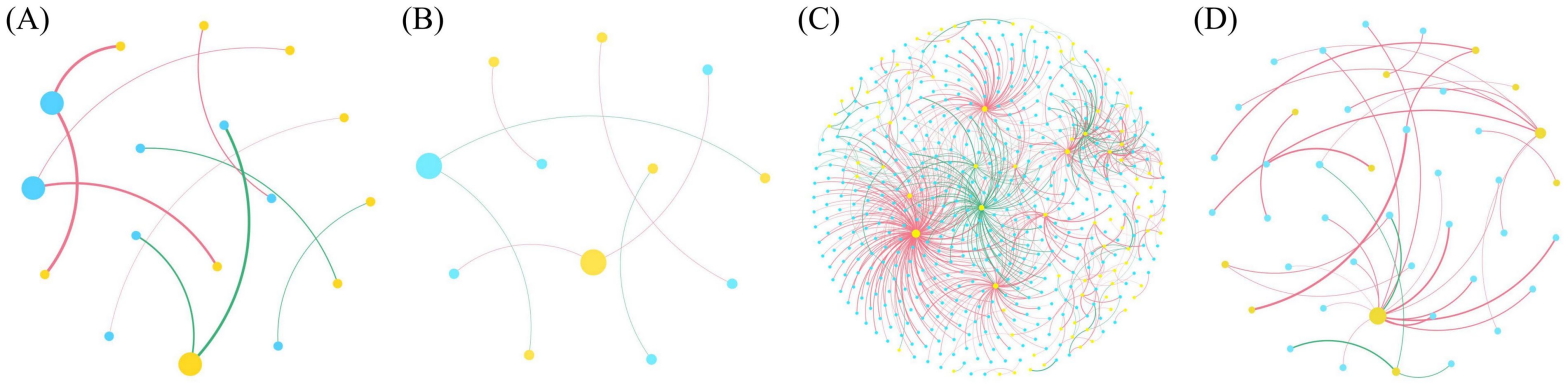
Pollen bacteria–fungi co-occurrence interaction networks under different treatments. **(A)** Self_open; **(B)** Self_bagging; **(C)** Cross_open; **(D)** Cross_bagging. “Self” denotes the self-pollinated type, and “Cross” denotes the cross-pollinated type. In the co-occurrence networks, nodes represent genera, and node size is proportional to node degree, with larger nodes indicating higher connectivity. Node colors denote different network modules; only the top 10 modules are displayed, while smaller modules are shown in grey. Edges represent significant correlations, with edge colors indicating positive (red) or negative (green) associations. Edge width is proportional to the absolute value of the correlation coefficient, with thicker edges indicating stronger correlations.

In bacteria-fungi interaction networks, all four networks displayed modular structures (modularity > 0.61). The two self-pollinated treatments and the cross-pollinated bagging treatment formed relatively small interaction networks, each comprising fewer than 50 nodes and 40 edges (Figure 4; Supplementary Table S4). In contrast, the cross-pollinated open treatment generated a markedly more expanded interaction network compared with the other networks. This interaction network exhibits a range of distinct topological features, including the highest average degree, the largest network diameter, the longest average path length, and the greatest number of core taxa, together with the lowest network density, indicating that a pronounced restructuring of cross-domain interaction architecture under the cross-pollinated open treatment.

### 3.4 Relationship between pollen remaining rate and predicted functions of core genera

Based on the bacteria–fungi interaction networks, a total of 90 core genera were identified. Notably, 88.89% of these core genera were identified in the cross-pollinated open network, whereas no core fungal genera were detected in the self-pollinated open network (Table 1). Among the 51 bacterial core genera, the majority belonged to Proteobacteria (19 genera), Firmicutes (14 genera), and Actinobacteriota (9 genera) (Supplementary Table S5). The 39 fungal core genera were mainly affiliated with Ascomycota (21 genera) and Basidiomycota (14 genera). The overall contributions of these core genera to pollen bacterial and fungal communities varied markedly among treatments (Table 1). In bacterial communities, the taxonomic relative abundances of core genera in both the self-pollinated open and bagging treatments were below 0.01%, and their corresponding predicted functional relative abundances were below 10% (Table 1; Supplementary Table S6). In contrast, in the cross-pollinated open treatment, the taxonomic and predicted functional relative abundances of bacterial core genera reached 37 and 25.49%, respectively. Similar but more pronounced patterns were observed in fungal communities: core genera in the cross-pollinated open treatment accounted for as much as 87.72% of the total fungal relative abundance and exhibited a wide range of trophic modes, whereas the relative abundances of fungal core genera in other treatments were consistently low (<3%) (Table 1; Supplementary Table S7). From both taxonomic and predicted functional perspectives, core genera under self-pollinated treatments were primarily low-abundance taxa (<1%). In contrast, under the cross-pollinated open treatment, core genera—particularly fungal core taxa—accounted for substantially higher proportions of total relative abundance and predicted functional potential.

**Table 1:** Contribution of core bacterial and fungal genera to the pollen microbiome under different pollination treatments.

Group		Core genus numbers	Core taxonomic relative abundance (%)	Core functional relative abundance (%)
Bacteria	Self_open	2	0.005 ± 0.006	3.63 ± 0.85
	Self_bagging	1	0.007 ± 0.006	8.17 ± 5.43
	Cross_open	46	37.00 ± 7.56	25.49 ± 5.71
	Cross_bagging	2	0.74 ± 0.49	2.20 ± 0.53
Fungi	Self_bagging	1	0.13 ± 0.08	0.13 ± 0.08
	Cross_open	34	87.72 ± 4.95	87.72 ± 4.95
	Cross_bagging	4	2.67 ± 2.45	2.67 ± 2.45

Core genera were identified based on interaction networks. “Self” denotes the self-pollinated type, and “Cross” denotes the cross-pollinated type. No core fungal genera were detected in the Self_open group. Data are presented as mean ± standard error (SE). Because each fungal genus corresponds to a single trophic mode as defined by FUNGuild, the relative abundance of predicted functions is numerically identical to the taxonomic relative abundance. Detailed predicted functional classifications within each group are provided in Supplementary Tables S6, S7.

We further focused on the open treatment to examine the relationship between pollen remaining rate and the predicted functions of core taxa. Under the open treatment, pollen remaining rates differed significantly among the four plant species, following the order: MC > SM > LA > CA, with CA exhibiting the greatest pollen loss (Table 2). At the species level, pollen remaining rate showed a positive association with the relative abundance of predicted functions of core bacterial genera (*ρ* > 0), indicating that plant species with higher pollen remaining rates tended to harbor higher relative abundances of predicted functions associated with core bacterial taxa in their pollen microbiomes. The pollen remaining rate of MC was substantially higher than that of LA, reflecting a very strong species effect (Cohen’s *d* = −3.07). In contrast, the relative abundance of predicted functions of core fungal genera differed only slightly between LA and MC (Cohen’s *d* = −0.33) and did not vary in parallel with the pronounced differences in pollen remaining rates (Table 3). Collectively, pollen remaining rate showed a positive association with predicted functions of core bacterial genera but not with those of core fungal genera.

**Table 2:** Pollen remaining rates of the four plant species under the open treatment.

Plant species	Pollen remaining rate (%)	Significance
CA	64.94 ± 1.01	c
SM	90.04 ± 1.67	b
LA	83.01 ± 2.87	b
MC	97.23 ± 0.59	a

Significance differences are indicated by the letter notation method, *p* < 0.05.

**Table 3:** Relationship between pollen remaining rate and predicted functions of microbial core genera under the open treatment.

Bacteria		Fungi		
**Metric**	Value	Metric	Pollen remaining rate (%)	Core genus function (%)
Spearman’s *ρ* (median)	0.40	Mean (LA)	83.01	85.02
Spearman’s *ρ* (mean)	0.53	Mean (MC)	97.22	90.41
95% CI of *ρ*	0.20–0.80	Difference (LA − MC)	−14.22	−5.39
Number of species	4	Cohen’s *d* (LA − MC)	−3.07	−0.33
Bootstrap iterations	5,000	Replicates per species	5	5

All analyses were conducted using open-pollinated samples only. “*ρ*” denotes the Spearman’s rank correlation coefficient. Bacterial functions were evaluated using a bootstrap-based species-level Spearman correlation analysis. Fungal functions were compared between the two cross-pollinated species (LA and MC) using effect size estimates because core fungal genera were not detected in self-pollinated species.

## 4 Discussion

### 4.1 Effects of insect visitation and host identity on pollen microbial community structure

In plant–pollinator interactions, microorganisms are increasingly recognized as indispensable participants (Steffan et al., 2024). A growing body of research has demonstrated that nectar-associated microorganisms can play pivotal roles in plant–pollinator interactions (Schaeffer and Irwin, 2014; Rering et al., 2018). At the same time, the composition and structure of nectar microbial communities are shaped by multiple factors, including insect visitation activity, pollinator identity, and host plant identity (Fridman et al., 2012; de Vega et al., 2021). In contrast, although pollen-associated microorganisms are also known to have important ecological functions (Zasloff, 2017), our understanding of the community structure and driving mechanisms of pollen microbiomes remains limited.

Our results showed that pollen microbial community composition differed significantly among host plant species under the bagging and open treatments (Figure 1; Supplementary Figure S1), indicating that both insect visitation and host plant identity may be involved in shaping pollen microbial communities. Previous studies have demonstrated that host plant identity is a key factor influencing pollen-associated microbial communities (Ambika Manirajan et al., 2018; Khalaf et al., 2023). Although direct experimental evidence manipulating insect visitation to assess its effects on pollen microbiomes remained lacking, pronounced differences among plant species with different pollination types have provided indirect support for the potential role of insect visitation in pollen microbiome assembly (Ambika Manirajan et al., 2018, 2019). In this study, OTU-level analyses further strengthened this pattern. The higher proportions of shared OTUs in bacterial communities and the markedly elevated proportions of unique fungal OTUs under open treatment, particularly in cross-pollinated host plants, suggest differential sensitivity of bacteria and fungi to insect-mediated processes. At the phylum level, Proteobacteria and Ascomycota were the most abundant phyla in bacterial and fungal communities (Figure 2), respectively, consistent with the findings of Ambika Manirajan et al. (2018). Moreover, shifts in the relative abundances of dominant phyla and the appearance or disappearance of several low-abundance phyla under the open treatment further suggested that insect visitation may influence pollen microbiome assembly through selective transport and host-mediated filtering processes. Similarly, Francis et al. (2023) demonstrated the synergistic roles of insect visitation and host identity in structuring nectar microbial communities. Taken together, by integrating the results of our bagging experiment with existing evidence, we infer that insect visitation and host plant identity jointly contribute, at least in part, to the observed variation in pollen microbial communities.

Differential responses of bacterial and fungal communities to environmental change have been widely documented in soil ecosystems and are largely attributed to niche differentiation driven by resource competition (Wang and Kuzyakov, 2024). A similar pattern was observed in pollen-associated microbial communities in the present study. Under the combined influences of insect visitation and host plant identity, bacterial and fungal communities exhibited markedly contrasting response patterns. Bacterial communities showed relatively stable overall structure and *α* diversity across treatments and host species, whereas fungal communities were considerably more sensitive to insect visitation and host plant differences (Figures 1–3; Supplementary Table S2). This may be associated with the higher sensitivity of fungal communities to insect-mediated disturbances that alter the floral microenvironment and resource conditions among host plants, changes that are more likely to induce variation in fungal assemblages. Previous studies have reported that bacterial communities associated with nectar, pistils, and stamens tended to exhibit greater stability, whereas fungal communities were more strongly shaped by host plant identity or geographic distribution (de Vega et al., 2021; Qian et al., 2021). Such differential responses may be closely linked to inherent differences between bacteria and fungi in ecological strategies, dispersal pathways, and environmental tolerance (Fu et al., 2024). Compared with bacteria, fungal communities often displayed stronger host selectivity and a greater dependence on biotic interactions, making their dispersal and colonization processes more susceptible to insect visitation and host plant traits (de Vega et al., 2021; Qian et al., 2021; Hietaranta et al., 2023). Consistent with this view, fungal communities in our study exhibited more pronounced composition shifts and α diversity differences under the open treatment, suggesting that insect visitation may accelerate fungal community differentiation among host plants through selective transport, repeated contact, and host-mediated filtering processes.

Therefore, although insect visitation and host plant identity jointly influence the composition and diversity of pollen microbial communities, the contrasting patterns of community stability and response intensity between bacteria and fungi reflect their distinct functional roles and assembly mechanisms within the pollen niche. Such differential responses may further shape the spatial heterogeneity and functional differentiation of pollen-associated microbial communities, thereby contributing in different ways to the dynamics of plant–pollinator–microbe interactions.

### 4.2 Effect of insect visitation on pollen microbial co-occurrence network

In multispecies ecological communities, complex interactions among species are ubiquitous, and co-occurrence networks derived from these interactions often provide a more comprehensive understanding of community responses to external disturbances than species composition alone (Gao et al., 2022). As highlighted by Vannette (2020), interactions among microorganisms are one of the key mechanisms driving variation in floral microbiomes. Therefore, after clarifying the effects of insect visitation and host plant identity on community structure, analyzing pollen microbiomes from a co-occurrence network perspective can help to elucidate their potential assembly mechanisms. Across 14 highly heterogeneous environments worldwide, the proportion of negative interactions in microbial co-occurrence networks has been reported to range from 1.9 to 48.9% (Ma et al., 2020). In contrast, the proportion of negative interactions in the pollen microbial networks in this study ranged only from 0 to 2.2%, which is substantially lower than that observed in most natural environments (Supplementary Table S3). This result suggests that within low-heterogeneity microhabitats such as pollen, strong environmental filtering associated with host plant identity and insect visitation favors the successful colonization and persistence of bacterial and fungal taxa, which are more likely to adopt cooperative or coexisting strategies rather than predominantly competitive relationships. From ecological and network-ecological perspectives, network connectivity and modularity are commonly used to characterize the strength of species interactions and the degree of niche differentiation within communities (Olesen et al., 2007; Ma et al., 2020). In single networks, the open treatment significantly altered the structure of both bacterial and fungal pollen networks in cross-pollinated plants; however, bacteria and fungi exhibited markedly different response patterns to insect visitation (Supplementary Figure S2, Supplementary Table S3). Under open treatment, bacterial networks showed increased connectivity and reduced modularity, indicating that insect visitation strengthens interactions among bacterial taxa within the community, while driving the community structure toward a more generalized state. In contrast, fungal networks under open treatment exhibited decreased connectivity and increased modularity, consistent with weakened inter-taxon connectivity and potentially enhanced niche differentiation within the fungal community. In addition, differences between fungal and bacterial single networks in clustering coefficient, network diameter, average path length, and number of modules may indicate more pronounced structural compartmentalization and niche differentiation within fungal communities, potentially reflecting greater sensitivity to host- or insect-mediated filtering processes (Faust and Raes, 2012; Ma et al., 2020). This pattern is highly consistent with the results in Section 4.1, where fungal community composition and *α* diversity displayed greater differentiation under open treatment. In self-pollinated plants, the effects of insect visitation on bacterial and fungal pollen networks were not consistent, and this discrepancy may be more strongly constrained by host plant identity or arise from trade-offs between bacteria and fungi in resource utilization and colonization processes.

Based on these results, we treated the pollen microbiome as an integrated system and constructed bacteria–fungi interaction networks (Figure 4). The results showed that the modularity values of all four interaction networks exceeded 0.61 (Supplementary Table S4). At the island scale, modularity values of empirical floral microbial networks have been reported to range from 0.22 to 0.83 (Qian et al., 2021), whereas at the global scale, microbial co-occurrence networks across diverse environments typically exhibit modularity values concentrated between 0.1 and 0.3 (Ma et al., 2020). These empirical comparisons indicate that pollen microbial networks exhibit pronounced modular structures rather than random assembly. We further found that interaction networks of self-pollinated plants were relatively small and weakly connected under both open and bagging treatments, whereas interaction networks of cross-pollinated plants—particularly under open treatment—showed marked expansion in network size and connectivity (Figure 4; Supplementary Table S4). This pattern suggests that insect visitation and pollination type are associated with restructuring of pollen microbial interaction architecture. Compared with single networks, the proportion of negative correlations in the interaction networks increased substantially (11.4–57.1%), indicating that microorganisms may adopt different ecological strategies within communities versus between communities. However, Ambika Manirajan et al. (2018) reported a much higher proportion of positive interactions (>75%) in pollen microbial interaction networks. This discrepancy likely arises because the interaction networks in the present study emphasize inter-community linkages and exclude intra-community interactions, thereby amplifying the relative contribution of negative interactions between microbial groups. In small-scale, resource-limited pollen microhabitats, microbial taxa that successfully colonize after strong environmental filtering may form relatively stable cooperative relationships within communities, allowing them to share ecological niches (Ma et al., 2020). In contrast, between communities, differences in resource utilization and competitive interactions among microorganisms may promote stronger niche differentiation, the extent of which is further modulated by host plant pollination type.

Overall, these findings indicate that insect visitation and host pollination type reshape the network structure of pollen microbiomes by altering patterns of interspecific interactions. Insect visitation is associated with the expansion and restructuring of pollen microbial networks in cross-pollinated plants, while driving bacterial networks toward a more generalized organization and fungal networks toward greater differentiation. These contrasting network-level responses further confirm that insect visitation and pollination type jointly shape pollen-associated bacterial and fungal communities, with bacterial communities exhibiting more integrated network organization overall, whereas fungal communities displayed stronger compositional differentiation and modular reorganization in response to disturbance.

### 4.3 Effect of insect visitation on core taxa and their predicted functions in pollen microbes

During insect visitation, pollinators facilitate pollen transfer among plants by carrying and depositing pollen, and their pollen-carrying capacity is closely associated with floral traits as well as the morphology and behavior of visiting insects (Moquet et al., 2017). Our results showed that cross-pollinated (insect-pollinated) plants exhibited higher mean pollen remaining rates than wind-pollinated (self-pollinated) plants, and that significant differences in pollen remaining rates were also observed among host plant species within the same pollination type. These findings indicate that both pollination type and host plant identity jointly influence pollen transfer processes. Compared with the pollen removal rate (40%) reported for the wild plant *Salix phylicifolia* (Hietaranta et al., 2023), the corresponding pollen removal rates (calculated as 100%—pollen remaining rate) of the two cross-pollinated plant species in this study (16.99 and 2.77%) were substantially lower. This difference may be attributed to reduced pollinator diversity and activity intensity in agricultural ecosystems, as well as stronger anthropogenic disturbances.

In microbial communities, only a small subset of taxa typically plays a disproportionate role in maintaining community structure and ecological functions; such taxa are commonly referred to as core or keystone taxa (Olesen et al., 2007; Banerjee et al., 2018). Based on co-occurrence network analysis, a total of 90 core genera were identified in the pollen microbiome in this study. Of these, 46.67% belonged to the bacterial phyla Proteobacteria, Firmicutes, and Actinobacteriota. These phyla are ubiquitous in natural ecosystems and have frequently been identified as core taxa in microbial co-occurrence networks across diverse environments (Ma et al., 2020). In addition, 38.89% of the core genera were affiliated with the fungal phyla Ascomycota and Basidiomycota, both of which encompass numerous yeast taxa, which are important components of floral-associated microbiomes, including nectar and pollen (Vannette, 2020). These results are largely consistent with previous studies on floral microbiomes, which have reported that core taxa are predominantly affiliated with Proteobacteria, Bacteroidetes, and Ascomycota (Ambika Manirajan et al., 2018). However, our findings contrast with a study that did not identify core fungal taxa in floral microbial networks (Qian et al., 2021). This discrepancy suggests that the composition of core taxa may be jointly influenced by host plant identity, the composition of visiting pollinators, and environmental conditions. We further found that in cross-pollinated host plants, insect visitation was significantly associated with an increased number of core taxa in pollen microbial communities, and that core genera contributed substantially to overall community structure and predicted functional profiles. Similar to the contributions reported by Ambika Manirajan et al. (2018), who found that core bacterial and fungal genera accounted for approximately 27 and 79% of the relative abundance in pollen microbiomes, respectively, core bacterial genera in our study accounted for 37% of the taxonomic relative abundance and 25.49% of the predicted functional relative abundance, while core fungal genera contributed as much as 87.72% of the total relative abundance. Moreover, insect visitation significantly altered the trophic modes of core fungal genera in the pollen microbiome of cross-pollinated plants, with the relative abundances of pathotrophs, saprotrophs, and unassigned trophic modes increasing significantly (Supplementary Table S7). These results, based on database-derived predictive functional annotations, suggest that insect visitation may facilitate the enrichment of fungal taxa with diverse ecological strategies by introducing fungi from multiple sources and modifying pollen microenvironmental conditions. Some of these fungi were assigned to trophic modes with potential pathogenic or saprotrophic attributes according to database classifications, which may contribute to increased inferred functional diversity of fungal core taxa within the pollen microbial community.

We further quantified the relationship between core taxa and insect visitation intensity by using pollen remaining rate as a proxy for pollen loss. At the species level, pollen remaining rate showed a synchronous trend with shifts in the relative abundance of predicted functions of core bacterial taxa, whereas the predicted functions of core fungal taxa did not respond synchronously to pollen removal. These results suggest that bacterial and fungal core taxa may adopt different response strategies to pollen loss induced by insect visitation. Specifically, bacterial core taxa, similar to the overall bacterial community, appear to be relatively stable, with changes in predicted functions tracking trends in pollen remaining rate. In contrast, fungal core taxa, consistent with the higher sensitivity of the fungal community, exhibited more complex functional responses. Although previous studies have reported consistent and significant responses of bacterial and fungal richness in inflorescences to pollen loss (Hietaranta et al., 2023), our findings indicate that the response of core taxa and the overall community of bacteria and fungi to external disturbances may differ, and the underlying mechanisms require further experimental validation. Overall, analyses based on core taxa and their predicted functions further corroborate the conclusions presented in Sections 4.1 and 4.2, namely that under the combined effects of host plant identity and insect visitation, pollen-associated bacterial communities remain relatively stable, whereas fungal communities are more sensitive to disturbance.

Although this study provides new insights into how insect visitation and host plant identity shape pollen microbial communities, several limitations should be acknowledged. The study was conducted at a single site during one flowering season, but broader spatial and temporal variation may influence pollen microbiome assembly. Pooling multiple flowers per sample facilitated community-level analyses but may have masked fine-scale variation among individual flowers. In addition, although no-template PCR controls were included, negative extraction controls and mock community standards were not incorporated, which may limit the assessment of low-level contamination and amplification bias. OTU-based clustering was applied to examine community-level ecological patterns; however, ASV-based approaches may provide higher taxonomic resolution and improved reproducibility. Furthermore, because microbiome data are inherently compositional, interpretations based on relative abundance should be made cautiously. Future studies incorporating multi-site and multi-season sampling, controlled manipulations of pollinator identity and visitation frequency, more comprehensive quality control designs, and compositional or ASV-based analytical frameworks will further refine our understanding of pollen microbiome assembly and its ecological consequences.

## 5 Conclusion

In this study, we systematically evaluated the effects of insect visitation and host plant identity on crop pollen microbiomes in an agricultural ecosystem using bagging and open treatments. Our results demonstrate that insect visitation and pollination type jointly shape pollen microbial communities, but bacterial and fungal communities exhibit markedly different response patterns. Bacterial communities remain relatively stable overall, whereas fungal communities are more sensitive to insect visitation and host plant identity. In cross-pollinated plants, insect visitation reshapes the pollen microbial interaction networks, promoting more integrated organization in bacterial networks while enhancing modularity and niche differentiation in fungal networks. Compared with bacteria, fungal communities exhibit more pronounced changes in community structure, composition, and diversity following insect visitation. In addition, insect visitation increases the relative contribution of fungal core taxa to community structure and predicted functions. Overall, this study reveals differential ecological strategies of pollen-associated bacterial and fungal communities in response to external disturbances during pollination, highlights the microbial dimension of pollination processes, and provides a novel ecological perspective for understanding crop reproductive ecology and plant–pollinator–microbe interactions.

## Data Availability

The raw sequencing data generated in this study have been deposited in the NCBI Sequence Read Archive (SRA) under BioProject accession number PRJNA1426584 (https://www.ncbi.nlm.nih.gov/bioproject/PRJNA1426584). Detailed sequencing statistics and OTU abundance tables are provided in the Supplementary material.

## Footnotes

1
https://www.arb-silva.de/

2
http://unite.ut.ee/

3
http://huttenhower.sph.harvard.edu/galaxy

4
http://www.funguild.org/
