# Supplementary material for: Differential responses of crop pollen microbial communities to insect visitation and host identity: fungi are more sensitive than bacteria
Source: Front Microbiol. 2026 Mar 23;17:1789970. doi: 10.3389/fmicb.2026.1789970 (PMC13050911; doi:10.3389/fmicb.2026.1789970)
Supplement: Supplementary file 1 [file Data_Sheet_1.docx]

Supplementary Material

# Supplementary Figures and Tables

## Supplementary Figures

**
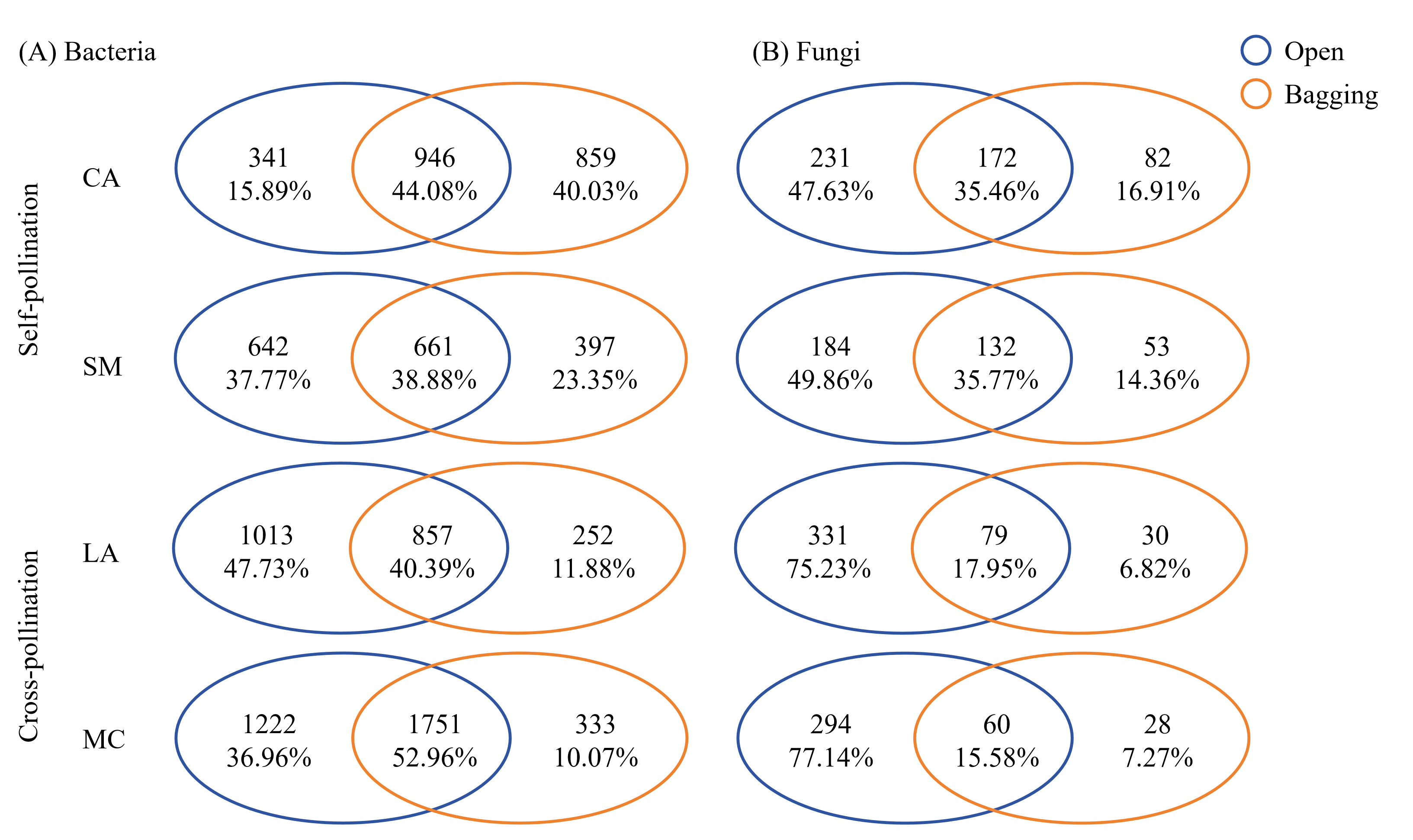
**

**Figure S1.** Numbers and proportions of shared and unique OTUs in pollen bacterial and fungal communities. CA, *Capsicum annuum* L.; SM, *Solanum melongena* L.; LA, *Luffa aegyptiaca* Mill.; MC, *Momordica charantia* L..


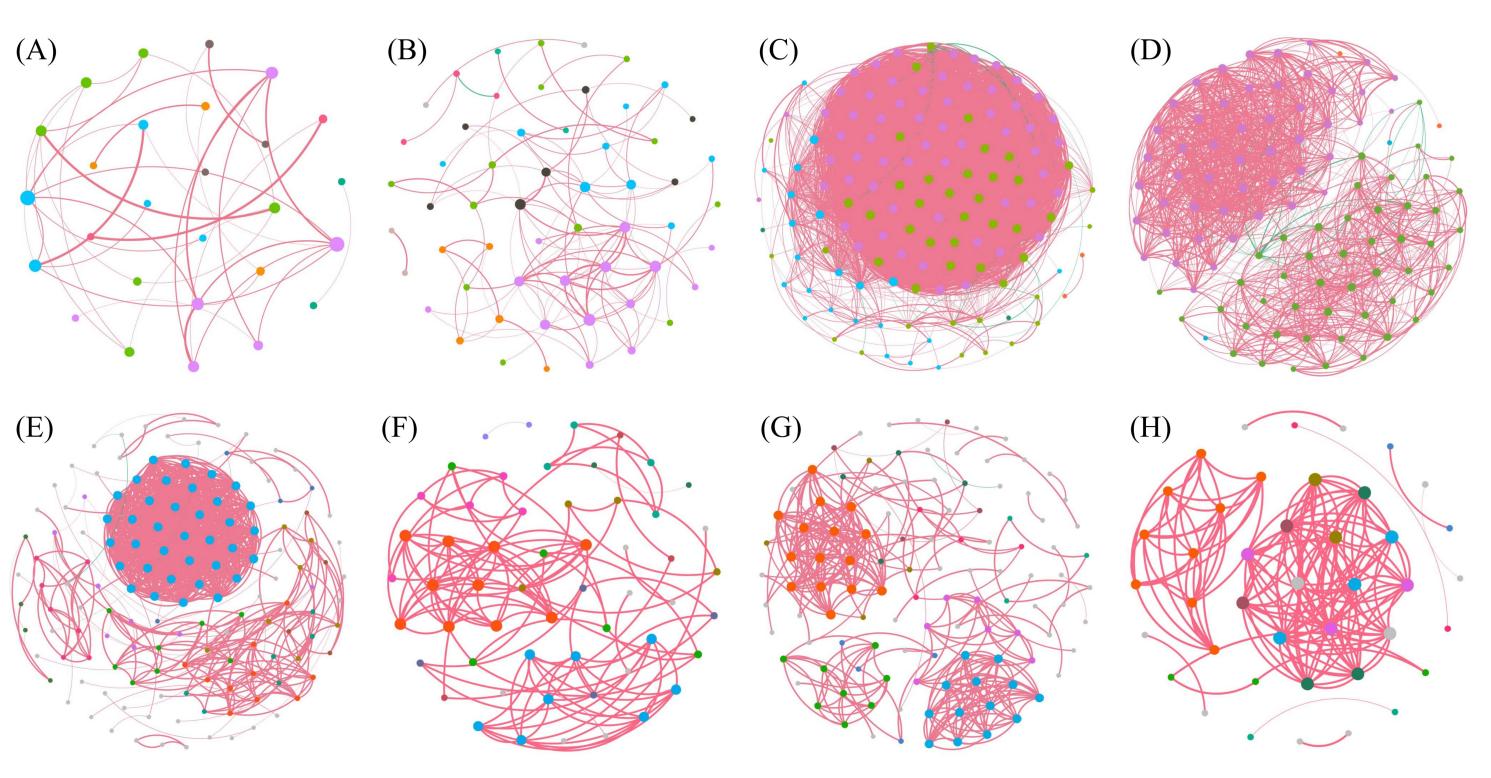


**Figure S2.** Co-occurrence networks of pollen bacteria and fungal communities at different treatments and pollination types. A, Bacteria_Self_open; B, Bacteria_Self_bagging; C, Bacteria_Cross_open; D, Bacteria_Cross_bagging; E, Fungi_Self_open; F, Fungi_Self_bagging; G, Fungi_Cross_open; H, Fungi_Cross_bagging. Detailed information on nodes and edges in the co-occurrence networks is shown in Figure 4.

## Supplementary Tables

**Table S1.** Detailed sequencing information for each sample, including SRA accession numbers, raw read counts, quality-filtered reads, read length statistics, and valid reads after chimera and host-sequence removal for bacterial (16S rRNA) and fungal (ITS) datasets.

| Sample | SRA accession number | Treatment | Sequencing strategy | Raw reads pairs | Clean read pairs | Mean length of clean reads | Valid reads (after chimera removal) | Valid reads (after host removal) |
| --- | --- | --- | --- | --- | --- | --- | --- | --- |
| MC1.16S | SRR37316144 | open | PE300 | 89935 | 84641 | 375.64664 | 71469 | 70267 |
| MC2.16S | SRR37316143 | open | PE300 | 71652 | 67265 | 376.40254 | 57344 | 56985 |
| MC3.16S | SRR37316110 | open | PE300 | 77511 | 73493 | 376.61709 | 66345 | 66092 |
| MC4.16S | SRR37316109 | open | PE300 | 78743 | 74083 | 376.43926 | 67143 | 65963 |
| MC5.16S | SRR37316108 | open | PE300 | 75058 | 71366 | 376.55526 | 66674 | 66583 |
| MC6.16S | SRR37316107 | bagging | PE300 | 15368 | 14587 | 375.55776 | 12317 | 5877 |
| MC7.16S | SRR37316106 | bagging | PE300 | 59772 | 53024 | 376.92006 | 48105 | 44137 |
| MC8.16S | SRR37316105 | bagging | PE300 | 56995 | 50429 | 375.51738 | 46034 | 44847 |
| MC9.16S | SRR37316104 | bagging | PE300 | 58603 | 50080 | 376.85146 | 48193 | 48159 |
| MC10.16S | SRR37316103 | bagging | PE300 | 52472 | 50258 | 377.41104 | 49660 | 48286 |
| CA1.16S | SRR37316090 | open | PE300 | 80362 | 77107 | 378.90941 | 75416 | 75410 |
| CA2.16S | SRR37316089 | open | PE300 | 79743 | 76139 | 377.75322 | 74163 | 74157 |
| CA3.16S | SRR37316088 | open | PE300 | 77693 | 74198 | 377.26371 | 72479 | 72465 |
| CA4.16S | SRR37316087 | open | PE300 | 68753 | 65708 | 377.19998 | 63116 | 63101 |
| CA5.16S | SRR37316086 | open | PE300 | 77853 | 74581 | 377.70077 | 72898 | 72784 |
| CA6.16S | SRR37316085 | bagging | PE300 | 70210 | 67000 | 376.63084 | 61673 | 61357 |
| CA7.16S | SRR37316084 | bagging | PE300 | 71237 | 68345 | 379.31855 | 66572 | 66564 |
| CA8.16S | SRR37316083 | bagging | PE300 | 75129 | 71821 | 377.48693 | 70121 | 70094 |
| CA9.16S | SRR37316082 | bagging | PE300 | 64812 | 61046 | 377.34381 | 59919 | 59880 |
| CA10.16S | SRR37316081 | bagging | PE300 | 76279 | 72721 | 376.99975 | 71042 | 71033 |
| SM1.16S | SRR37316101 | open | PE300 | 88342 | 85014 | 376.91888 | 84190 | 84186 |
| SM2.16S | SRR37316100 | open | PE300 | 66080 | 62623 | 377.08115 | 60632 | 60545 |
| SM3.16S | SRR37316099 | open | PE300 | 67901 | 64542 | 376.88962 | 63369 | 63342 |
| SM4.16S | SRR37316098 | open | PE300 | 85204 | 81503 | 374.8836 | 76715 | 76712 |
| SM5.16S | SRR37316097 | open | PE300 | 74209 | 71073 | 373.70191 | 70016 | 69988 |
| SM6.16S | SRR37316096 | bagging | PE300 | 52803 | 50690 | 376.50097 | 46860 | 40056 |
| SM7.16S | SRR37316095 | bagging | PE300 | 79940 | 76555 | 376.91155 | 74034 | 73982 |
| SM8.16S | SRR37316094 | bagging | PE300 | 60880 | 56653 | 376.74646 | 55235 | 55101 |
| SM9.16S | SRR37316093 | bagging | PE300 | 74953 | 71871 | 377.01167 | 70531 | 70527 |
| SM10.16S | SRR37316092 | bagging | PE300 | 54874 | 52751 | 377.01892 | 49297 | 47152 |
| LA1.16S | SRR37316146 | open | PE300 | 64813 | 56079 | 375.93447 | 52614 | 52574 |
| LA2.16S | SRR37316145 | open | PE300 | 75151 | 72012 | 371.16233 | 69381 | 69364 |
| LA3.16S | SRR37316102 | open | PE300 | 58374 | 54277 | 376.83921 | 49008 | 48796 |
| LA4.16S | SRR37316091 | open | PE300 | 85820 | 82591 | 377.06996 | 79271 | 79270 |
| LA5.16S | SRR37316080 | open | PE300 | 75744 | 72317 | 377.01877 | 68541 | 68407 |
| LA6.16S | SRR37316133 | bagging | PE300 | 86298 | 82717 | 377.1028 | 76849 | 76848 |
| LA7.16S | SRR37316122 | bagging | PE300 | 62526 | 56719 | 377.00453 | 52672 | 52526 |
| LA8.16S | SRR37316111 | bagging | PE300 | 97807 | 93723 | 376.89209 | 90581 | 90581 |
| LA9.16S | SRR37316068 | bagging | PE300 | 81743 | 78244 | 376.88561 | 76849 | 76847 |
| LA10.16S | SRR37316067 | bagging | PE300 | 79449 | 76236 | 377.06658 | 75569 | 75567 |
| MC1.ITS | SRR37316132 | open | PE300 | 120606 | 117926 | 306.29422 | 117809 | 117809 |
| MC2.ITS | SRR37316131 | open | PE300 | 173617 | 170310 | 305.86456 | 170134 | 170134 |
| MC3.ITS | SRR37316130 | open | PE300 | 62011 | 60748 | 300.57283 | 60669 | 60669 |
| MC4.ITS | SRR37316129 | open | PE300 | 50494 | 49457 | 299.99616 | 49391 | 49391 |
| MC5.ITS | SRR37316128 | open | PE300 | 72659 | 71193 | 295.47446 | 71122 | 71122 |
| MC6.ITS | SRR37316127 | bagging | PE300 | 57244 | 55983 | 288.99584 | 55950 | 55950 |
| MC7.ITS | SRR37316126 | bagging | PE300 | 44114 | 43216 | 313.71541 | 43184 | 43184 |
| MC8.ITS | SRR37316125 | bagging | PE300 | 52574 | 51718 | 313.77528 | 51681 | 51681 |
| MC9.ITS | SRR37316124 | bagging | PE300 | 44640 | 43802 | 313.29859 | 43780 | 43780 |
| MC10.ITS | SRR37316123 | bagging | PE300 | 43342 | 42397 | 312.95445 | 42365 | 42365 |
| CA1.ITS | SRR37316078 | open | PE300 | 75748 | 73224 | 235.34617 | 73219 | 73219 |
| CA2.ITS | SRR37316077 | open | PE300 | 80655 | 77927 | 250.91262 | 77807 | 77807 |
| CA3.ITS | SRR37316076 | open | PE300 | 78400 | 76396 | 235.0483 | 76307 | 76307 |
| CA4.ITS | SRR37316075 | open | PE300 | 74954 | 73450 | 238.96385 | 73311 | 73311 |
| CA5.ITS | SRR37316074 | open | PE300 | 72372 | 67166 | 235.64647 | 67127 | 67127 |
| CA6.ITS | SRR37316073 | bagging | PE300 | 42495 | 39330 | 238.34457 | 39307 | 39307 |
| CA7.ITS | SRR37316072 | bagging | PE300 | 70218 | 68984 | 273.23587 | 68844 | 68844 |
| CA8.ITS | SRR37316071 | bagging | PE300 | 70881 | 68065 | 257.78108 | 67891 | 67891 |
| CA9.ITS | SRR37316070 | bagging | PE300 | 65537 | 63805 | 246.91051 | 63756 | 63756 |
| CA10.ITS | SRR37316069 | bagging | PE300 | 76239 | 74740 | 247.72297 | 74250 | 74250 |
| SM1.ITS | SRR37316121 | open | PE300 | 70283 | 63782 | 267.6911 | 63747 | 63747 |
| SM2.ITS | SRR37316120 | open | PE300 | 71240 | 68222 | 249.63858 | 68164 | 68164 |
| SM3.ITS | SRR37316119 | open | PE300 | 74058 | 72286 | 269.41924 | 72247 | 72247 |
| SM4.ITS | SRR37316118 | open | PE300 | 65585 | 64179 | 280.41669 | 64118 | 64118 |
| SM5.ITS | SRR37316117 | open | PE300 | 73227 | 71435 | 264.87377 | 71360 | 71360 |
| SM6.ITS | SRR37316116 | bagging | PE300 | 65102 | 63893 | 294.93905 | 63790 | 63790 |
| SM7.ITS | SRR37316115 | bagging | PE300 | 59499 | 58399 | 297.93342 | 58386 | 58386 |
| SM8.ITS | SRR37316114 | bagging | PE300 | 92783 | 90944 | 263.02972 | 90879 | 90879 |
| SM9.ITS | SRR37316113 | bagging | PE300 | 109156 | 107154 | 261.55254 | 106888 | 106888 |
| SM10.ITS | SRR37316112 | bagging | PE300 | 94337 | 89907 | 271.63509 | 89852 | 89852 |
| LA1.ITS | SRR37316079 | open | PE300 | 68180 | 67044 | 265.37856 | 66895 | 66895 |
| LA2.ITS | SRR37316142 | open | PE300 | 48769 | 48147 | 279.95427 | 48116 | 48116 |
| LA3.ITS | SRR37316141 | open | PE300 | 67174 | 65994 | 279.6243 | 65875 | 65875 |
| LA4.ITS | SRR37316140 | open | PE300 | 55082 | 54067 | 277.3165 | 54001 | 54001 |
| LA5.ITS | SRR37316139 | open | PE300 | 59800 | 58476 | 279.80226 | 58420 | 58420 |
| LA6.ITS | SRR37316138 | bagging | PE300 | 57427 | 56192 | 270.36938 | 56149 | 56149 |
| LA7.ITS | SRR37316137 | bagging | PE300 | 64707 | 63621 | 280.02765 | 63591 | 63591 |
| LA8.ITS | SRR37316136 | bagging | PE300 | 57282 | 56558 | 280.9099 | 56533 | 56533 |
| LA9.ITS | SRR37316135 | bagging | PE300 | 57194 | 56160 | 273.1729 | 56141 | 56141 |
| LA10.ITS | SRR37316134 | bagging | PE300 | 55389 | 54126 | 266.91359 | 54111 | 54111 |

**Table S2.** Two-way ANOVA of pollen microbial diversity indices.

| **Diversity index** | **Factor** | **Treatment** | **Species** | **Treatment × Species** |
| --- | --- | --- | --- | --- |
|  | **df** | **1** | **3** | **3** |
| Bacteria Chao | *F* | 4.77 | 8.31 | 1.36 |
|  | *p* | 0.036 | <0.001 | 0.272 |
| Bacteria Shannon | *F* | 0.02 | 3.81 | 0.59 |
|  | *p* | 0.888 | 0.019 | 0.629 |
| Bacteria PD | *F* | 2.99 | 8.38 | 1.58 |
|  | *p* | 0.093 | <0.001 | 0.213 |
| Fungi Chao | *F* | 43.85 | 4.16 | 4.75 |
|  | *p* | <0.001 | 0.013 | 0.008 |
| Fungi Shannon | *F* | 1.25 | 34.62 | 1.4 |
|  | *p* | 0.271 | <0.001 | 0.261 |
| Fungi PD | *F* | 82.55 | 0.48 | 4.87 |
|  | *p* | <0.001 | 0.696 | 0.007 |

**Table S3.** Topological characteristics of pollen bacterial and fungal single co-occurrence networks across pollination types and treatments. “Self” denotes the self-pollinated type, and “Cross” denotes the cross-pollinated type.

| **Group** | **Bacteria** | | | | **Fungi** | | | |
| --- | --- | --- | --- | --- | --- | --- | --- | --- |
|  | **Self** | | **Cross** | | **Self** | | **Cross** | |
|  | **Open** | **Bagging** | **Open** | **Bagging** | **Open** | **Bagging** | **Open** | **Bagging** |
| Nodes | 27 | 56 | 130 | 101 | 143 | 55 | 113 | 41 |
| Edges | 38 | 107 | 4444 | 1293 | 826 | 120 | 295 | 151 |
| Average_degree | 2.815 | 3.821 | 68.369 | 25.604 | 11.552 | 4.364 | 5.221 | 7.366 |
| Clustering | 0.385 | 0.607 | 0.863 | 0.732 | 0.923 | 1 | 0.992 | 1 |
| Density | 0.108 | 0.069 | 0.53 | 0.256 | 0.081 | 0.081 | 0.047 | 0.184 |
| Diameter | 8 | 6 | 6 | 6 | 3 | 1 | 2 | 1 |
| Average_path_length | 3.289 | 2.75 | 1.643 | 2.179 | 1.035 | 1 | 1.003 | 1 |
| Modularity | 0.527 | 0.564 | 0.046 | 0.437 | 0.412 | 0.782 | 0.787 | 0.459 |
| Module numbers | 7 | 9 | 5 | 4 | 29 | 14 | 31 | 10 |
| Positive_edges | 38 | 106 | 4359 | 1264 | 823 | 120 | 294 | 151 |
| Negative_edges | 0 | 1 | 85 | 29 | 3 | 0 | 1 | 0 |
| Positive_edges_ratio | 1 | 0.991 | 0.981 | 0.978 | 0.996 | 1 | 0.997 | 1 |
| Negative_edges_ratio | 0 | 0.009 | 0.019 | 0.022 | 0.004 | 0 | 0.003 | 0 |
| Core taxa numbers | 4 | 9 | 26 | 19 | 29 | 11 | 24 | 8 |

**Table S4.** Topological characteristics of pollen bacterial-fungal interaction co-occurrence networks across pollination types and treatments. “Self” denotes the self-pollinated type, and “Cross” denotes the cross-pollinated type.

| **Group** | **Self_open** | **Self_bagging** | **Cross_open** | **Cross_Bagging** |
| --- | --- | --- | --- | --- |
| Nodes | 17 | 12 | 562 | 43 |
| Edges | 10 | 7 | 961 | 35 |
| Bacteria_nodes | 8 | 6 | 474 | 32 |
| Fungi_nodes | 9 | 6 | 88 | 11 |
| Average_degree | 1.176 | 1.167 | 3.42 | 1.628 |
| Density | 0.0735 | 0.1061 | 0.0061 | 0.0388 |
| Diameter | 2 | 2 | 17 | 4 |
| Average_path_length | 1.231 | 1.222 | 4.423 | 2.055 |
| Modularity | 0.84 | 0.776 | 0.616 | 0.731 |
| Module numbers | 7 | 5 | 24 | 9 |
| Positive_edges | 6 | 3 | 716 | 31 |
| Negative_edges | 4 | 4 | 245 | 4 |
| Positive_edges_ratio | 0.6 | 0.429 | 0.745 | 0.886 |
| Negative_edges_ratio | 0.4 | 0.571 | 0.255 | 0.114 |
| Core taxa numbers | 2 | 2 | 80 | 6 |

**Table S5.** Taxonomic classification of 80 core genera in the bacteria–fungi interaction networks.

| **Core genus** | **Phylum** | **Numbers** | **Group** |
| --- | --- | --- | --- |
| Bacteria | Proteobacteria | 19 | Cross_open, Cross_bagging, Self_open |
|  | Firmicutes | 14 | Cross_open, Cross_bagging |
|  | Actinobacteriota | 9 | Cross_open, Self_bagging |
|  | Verrucomicrobiota | 3 | Cross_open |
|  | Bacteroidota | 2 | Cross_open |
|  | Desulfobacterota | 1 | Cross_open |
|  | Entotheonellaeota | 1 | Cross_open |
|  | Gemmatimonadota | 1 | Cross_open |
|  | Myxococcota | 1 | Cross_open |
| Fungi | Ascomycota | 21 | Cross_open, Cross_bagging |
|  | Basidiomycota | 14 | Cross_open, Cross_bagging, Self_bagging |
|  | Fungi_phy_Incertae_sedis | 2 | Cross_open, Cross_bagging |
|  | Kickxellomycota | 1 | Cross_open |
|  | unclassified_k__Fungi | 1 | Cross_open |

**Table S6.** Detailed predicted functions of core bacterial genera and their relative proportions.

| **Bacteria group** | **Pathway level 1** | **Mean relative abundance±SE (%)** |
| --- | --- | --- |
| Self_open | Metabolism | 0.09±0.07 |
|  | Genetic information processing | 2.28±0.53 |
|  | Environmental information processing | 0.79±0.21 |
|  | Cellular processes | 0.35±0.09 |
|  | Organismal systems | 0.16±0.05 |
|  | Human diseases | 0.03±0.01 |
| Self_bagging | Metabolism | 5.18±3.41 |
|  | Genetic information processing | 1.19±0.80 |
|  | Environmental information processing | 1.10±0.72 |
|  | Cellular processes | 0.59±0.41 |
|  | Organismal systems | 0.03±0.02 |
|  | Human diseases | 0.09±0.07 |
| Cross_open | Metabolism | 16.00±3.55 |
|  | Genetic information processing | 6.42±1.53 |
|  | Environmental information processing | 1.98±0.43 |
|  | Cellular processes | 0.75±0.16 |
|  | Organismal systems | 0.22±0.05 |
|  | Human diseases | 0.12±0.03 |
| Cross_bagging | Metabolism | 1.43±0.35 |
|  | Genetic information processing | 0.38±0.10 |
|  | Environmental information processing | 0.25±0.06 |
|  | Cellular processes | 0.11±0.02 |
|  | Organismal systems | 0.01±0 |
|  | Human diseases | 0.01±0 |

**Table S7.** Detailed predicted functions of core fungal genera and their relative proportions.

| **Fungi group** | **Trophic mode** | **Mean relative abundance±SE (%)** |
| --- | --- | --- |
| Self_bagging | Pathotroph | 0.13±0.08 |
| Cross_open | Pathotroph | 1.80±0.86 |
|  | Saprotroph | 0.82±0.26 |
|  | Mixed trophic modes | 3.86±0.71 |
|  | Unassigned | 81.24±5.89 |
| Cross_bagging | Mixed trophic modes | 2.64±2.46 |
|  | Unassigned | 0.04±0.02 |
